# Water intake and intra-meal fluid consumption in relation to general and abdominal obesity of Iranian adults

**DOI:** 10.1186/s12937-020-00551-x

**Published:** 2020-05-02

**Authors:** Asma Salari-Moghaddam, Negar Aslani, Parvane Saneei, Ammar Hassanzadeh Keshteli, Parnaz Daneshpajouhnejad, Ahmad Esmaillzadeh, Peyman Adibi

**Affiliations:** 1 Department of Community Nutrition, School of Nutritional Sciences and Dietetics, Tehran University of Medical Sciences, P.O. Box 14155-6117, Tehran, Iran; 2 Department of Clinical Nutrition, Food Security Research Center, School of Nutrition and Food Science, Isfahan University of Medical Sciences, Isfahan, Iran; 3 Food Security Research Center, Isfahan University of Medical Sciences, Isfahan, Iran; 4 Department of Medicine, University of Alberta, Edmonton, Alberta Canada; 5 Integrative Functional Gastroenterology Research Center, Isfahan University of Medical Sciences, Isfahan, Iran; 6 Department of Community Nutrition, School of Nutrition and Food Science, Isfahan University of Medical Sciences, Isfahan, Iran; 7 Obesity and Eating Habits Research Center, Endocrinology and Metabolism Molecular-Cellular Sciences Institute, Tehran University of Medical Sciences, Tehran, Iran

**Keywords:** Water, Intra-meal fluid, General obesity, Abdominal obesity, Adults

## Abstract

**Objective:**

The aim of this study was to examine the association between whole-day water intake and intra-meal fluid consumption and odds of general and abdominal obesity among adults.

**Methods:**

This cross-sectional study was conducted among 7958 adults in Isfahan, Iran. Daily water consumption was assessed through the use of a pre-tested questionnaire by asking questions about the average number of glasses of water consumed in a day. Intra-meal fluid consumption was also analysed. Data regarding height, weight and waist circumference were collected using a validated self-administered questionnaire. Obesity was defined as body mass index ≥30 kg/m^2^, and abdominal obesity was defined as waist circumference >88 cm for women and >102 cm for men.

**Results:**

After taking potential confounders into account, individuals who were taking more than eight glasses of water in a day had 78% greater odds of general obesity (OR: 1.78; 95% CI: 1.08–2.94) compared with those who were taking less than two glasses of water. Individuals with much water intake had no significant greater odds of abdominal obesity. Compared with those who were consuming less than a glass of intra-meal fluids, subjects with 1–2 glasses of fluids between meals had 34% greater odds of general obesity (OR: 1.34; 95% CI: 1.04–1.59). Although subjects with greater intra-meal fluid intake had greater odds of abdominal obesity in crude model, this association became non-significant after adjustment for potential confounders (comparing > 4 glasses vs. ≤1 glass: OR: 1.65; 95% CI: 0.81–3.34).

**Conclusions:**

We observed that taking more than eight glasses of water in a day and consuming 1–2 glasses of fluids between meals was associated with greater odds of general obesity.

## Introduction

Obesity, characterized by excess adipose tissue, is highly prevalent in the world [1]. It is a major risk factor for mortality and morbidity [2]. In 2015, a total of 107.7 million children and 603.7 million adults were obese worldwide [3]. According to World Health Organization report, more than 1.9 billion adults ≥18 years were overweight worldwide in 2016; of them, over 650 million were obese [4]. The rapid increase in the prevalence of obesity makes the identification and modification of obesity risk factors as a priority.

In addition to the involvement of several factors in the etiology of obesity, water intake has been postulated to play a key role in the management of body weight. Water comprises about 60% of body weight [5], but the key role of water in health maintenance remains understudied. Recommendations to lose weight suggested drinking a glass of water before a main meal [6]. Such advice along with a hypocaloric diet has been resulted in a greater weight loss than a hypocaloric diet alone [6]. Replacing sugar-sweetened beverages with water and low caloric beverages has been resulted in a greater weight loss [7]. Findings from a systematic review revealed that much water intake was associated with a lower energy intake, and therefore, obesity prevention [5]. However, this is not the case in all published documents. Walleghen et al. reported that pre-meal water intake did not affect meal energy intake in young women [8]. It has also been hypothesized that water intake from foods, not from beverages, was associated with a lower body mass index (BMI) and waist circumference (WC) [9].

It seems that water drinking affects resting energy expenditure (REE), such that those with high water intake had higher REE than those with a low intake [10]. Most information on the water-weight associations came from clinical trials, which are beyond the habitual intake of water in routine life [6]. Observational studies linking water consumption to energy intake, weight and obesity are scarce [11, 12]. In a cross-sectional study, weight loss holders drank much water than those who failed to hold their lost weight [11]. Another longitudinal study showed greater reductions in WC and body weight when subjects drank more than 1 L water/day over 12 months [12].

In addition to the limited epidemiologic evidence, patterns of drinking water are different across the world. While in most western and developed countries, sweetened beverages are used to respond thirst; this is not the case in most developing countries, including the Middle-East. Water consumption ranks as a second drink after tea, the most popular drink, in these countries [13]. In addition to quantity of water intake, it has always been a question if intra-meal water intake can affect weight. The association of other types of fluids including sugar-sweetened beverages, coffee and tea consumption with obesity has been extensively examined in previous studies [14–16]. However, limited data are available on the association between whole-day water intake and intra-meal fluids consumption in relation to obesity. This study was, therefore, conducted to investigate the relationship between whole-day water intake and intra-meal fluids consumption and prevalence of general and abdominal obesity among a large group of Iranian adults.

## Methods and materials

### Study population

This study was done in the framework of the Study on the Epidemiology of Psychological, Alimentary Health and Nutrition (SEPAHAN) project, which was performed among a large group of adult populations in Isfahan, Iran. All participants were general adults who were working in 50 different health centres across Isfahan province. Comprehensive information about the study design and data collection methods have been published elsewhere [17]. Required data on anthropometric measures, socio-demographic information and dietary intakes along with physical activity were collected through the use of a pretested self-administered questionnaire. The questionnaire was sent to 10,087 adults aged 18–55 years and 8691 subjects returned the completed questionnaire (response rate: 86.16%). In the current study, participants whom total calorie intake was out of the range of 800–4200 kcal/day were excluded due to under- and over-reporting of energy intake. We also excluded subjects who lacked information on required variables. These exclusions resulted in 7958 adults for the analysis on general obesity and 6054 adults for the analysis on abdominal obesity. All participants provided signed informed written consent forms. The whole project of SEPAHAN was ethically approved by the Bioethics Committee of Isfahan University of Medical Sciences, Isfahan, Iran.

### Assessment of water intake

To examine total daily water intake in routine life, participants were requested to report the average number of glasses of water they usually consume in a day. The possible choices to this question were < 2, 2–5, 6–8 and > 8 glasses of water during the whole day. In addition, they were asked to report their usual intra-meal fluid intake (< 1, 1–2, 3–4 and > 4 glasses).

### Anthropometric assessment

Required information on weight, height and WC was collected through the use of a self-reported questionnaire. BMI was computed as weight in kilogram divided by height in meters squared. Obesity was defined as having BMI ≥30 kg/m^2^. Abdominal obesity was determined based on WC. We used the National Cholesterol Education Program (NCEP) guidelines for categorizing subjects in terms of their WC: normal (< 80 cm for women and < 94 cm for men), abdominally overweight (80–88 cm for women and 94–102 cm for men) and abdominally obese (> 88 cm for women and > 102 cm for men) [18].

The validity of self-reported data on height, weight and waist circumference was examined in a pilot study on 200 individuals from the same population. In the validation study, self-reported values of anthropometric indicators were assessed against the measured values. The correlation coefficients for self-reported weight, height and WC against their corresponding measured values were 0.95 (*P < 0.001*), 0.83 (*P < 0.001*) and 0.60 (*P < 0.001*), respectively. The correlation coefficient for calculated BMI from self-reported data and the one from measured values was 0.70 (*P < 0.001*). This information demonstrated that the self-reported data of anthropometric measures provide reasonably valid data for these indicators [19].

### Assessment of other variables

Dietary intakes of study participants were examined using a validated 106-item dish-based semi-quantitative food frequency questionnaire, as described elsewhere in detail [20]. Daily intakes of energy and macronutrients for each participant were estimated based on the US Department of Agriculture food-composition database, modified for Iranian foods. Required information on other variables including age, sex, education (university graduate and below that), smoking status (non-smoker, former smokers and current smokers), home ownership (owner/non-owner), breakfast consumption (skippers/non-skippers), quick eating of lunch (> 20 min/10–20 min/< 10 min), quick eating of dinner (> 20 min/10–20 min/< 10 min), meal regularity (never/occasionally/often/always) and number of meals (one/two/three) were collected using a pretested self-administered questionnaire. Those who were consuming breakfast < 4 times/week were defined as breakfast skippers, those who were consuming lunch and dinner in less than 10 min were defined as quick eaters of lunch and dinner and those who consumed all three main meals in a day were defined as having meal regularity. Physical activity levels of participants were assessed using the General Practice Physical Activity Questionnaire (GPPAQ) [21]. This questionnaire is a simple, four-level physical activity index (PAI) reflecting an individual’s current physical activity. In the current analysis, we classified participants into two categories: < 1 h/week (physically inactive) or ≥ 1 h/week (physically active).

### Statistical analysis

General characteristics of study participants across different categories of whole-day water intake and intra-meal fluid consumption were expressed as means ±SDs for continuous variables and percentages for categorical variables. To examine the differences across categories of whole-day water intake and intra-meal fluid consumption, we used one-way ANOVA for continuous variables and chi-square test for categorical variables. The multivariable-adjusted means for anthropometric measures across categories of whole-day water intake and intra-meal fluid consumption were computed and compared using ANCOVA. We also used binary logistic regression to estimate odds ratios (ORs) and 95% confidence intervals (CIs) for the presence of general and abdominal obesity across categories of whole-day water intake and intra-meal fluid consumption in crude and multivariable-adjusted models. Age (continuous), sex (male/female), total energy intake (continuous), education (diploma or under-diploma/university graduate), physical activity (< 1 h/week/≥1 h/week), marital status (married/single/divorced and widowed), home ownership (owner/non-owner), breakfast skipping (skippers/non-skippers), quick eating of lunch (> 20 min/10–20 min/< 10 min), quick eating of dinner (> 20 min/10–20 min/< 10 min), meal regularity (never/occasionally/often/always) and number of meals in a day (one/two/three) were controlled for in the multivariable-adjusted model. *P* for trends was determined by considering categories of whole-day water intake and intra-meal fluid consumption as ordinal variables in the logistic regression analysis. All statistical analyses were done using the Statistical Package for Social Sciences (version 20; SPSS Inc.). *P < 0.05* was considered as statistically significant.

## Results

Characteristics of study participants across categories of whole-day water intake and intra-meal fluid consumption are shown in Table 1. Participants who were taking > 8 glasses of water in a day, compared to those who consumed less than two glasses of water daily, were younger (*P < 0.001*), less likely to be quick eaters of lunch and dinner (*P < 0.001*), more likely to be males (*P < 0.001*), take all three main meals throughout the day (*P < 0.001*) and have regular meal pattern (*P < 0.001*). Compared with those who consumed less than a glass of fluid, participants who consumed > 4 glasses of fluid between meals were younger (*P = 0.01*), more likely to be males (*P < 0.001*), quick eaters of lunch and dinner (*P = 0.001*) and had low regularity in their meal patterns (*P < 0.001*). There was no significant difference in terms of dietary intakes across categories of whole-day water intake and intra-meal fluid consumption.

**Table 1:** Characteristics of study participants across categories of whole-day water intake and intra-meal fluid consumption^1^

	Whole-day water intake (glass/d)					Intra-meal fluid consumption (glass/d)				
	< 2	2–5	6–8	> 8	***P***-value^**2**^	< 1	1–2	3–4	> 4	P-value^**2**^
Age, y	38.0 ± 7.90	36.8 ± 8.23	36.01 ± 8.45	36.62 ± 8.98	< 0.001	37.0 ± 8.14	36.8 ± 8.43	35.0 ± 9.02	36.9 ± 6.55	0.01
Female, %	64.0	59.6	57.2	58.0	< 0.001	61.6	57.8	53.2	46.3	< 0.001
Married, %	81.5	83.0	84.3	83.6	0.29	82.5	83.8	81.5	94.3	0.09
University graduated, %	61.7	60.7	58.0	58.5	0.25	60.6	60.0	50.3	56.6	0.07
Home ownership, %	67.1	68.2	69.3	69.5	0.70	68.1	68.7	65.0	66.7	0.82
Physically active, %	34.1	33.3	34.2	32.0	0.86	32.9	34.6	40.7	32.0	0.16
Meal regularity (always), %	12.0	14.6	17.3	24.0	< 0.001	15.3	13.4	13.0	18.9	< 0.001
Number of meals (3 meals/day), %	60.3	68.6	71.0	68.3	< 0.001	67.1	67.5	70.2	62.2	0.72
Quick eating of lunch (< 10 min), %	20.2	14.0	13.0	17.4	< 0.001	15.6	15.5	15.3	18.9	0.001
Quick eating of dinner (< 10 min), %	28.3	22.0	20.6	21.8	< 0.001	24.6	21.5	18.6	25.7	< 0.001
Breakfast skippers, %	76.6	76.9	76.3	74.0	0.74	23.7	22.0	27.4	32.1	0.13
**Dietary intakes**										
Energy (kcal/d)	2340 ± 824	2373 ± 834	2388 ± 843	2382 ± 810	0.45	2362 ± 835	2377 ± 820	2415 ± 848	2355 ± 899	0.82
Protein (g/d)	87.4 ± 33.3	88.5 ± 33.9	89.2 ± 34.7	88.4 ± 32.7	0.53	88.1 ± 34	88.5 ± 33	91.8 ± 37.6	87.8 ± 35.6	0.59
Carbohydrate (g/d)	286.4 ± 116	290.1 ± 116	291.2 ± 117	290.2 ± 113	0.68	288.8 ± 116	290.3 ± 114	291.7 ± 121	287.4 ± 124	0.96
Fat (g/d)	97.7 ± 36.9	99.3 ± 37.9	100.1 ± 38.3	100.4 ± 37.3	0.31	98.8 ± 37.7	99.5 ± 37.2	101.6 ± 38.8	98.5 ± 42.5	0.75
Dietary fibre (g/d)	22.2 ± 9.8	22.5 ± 9.7	22.7 ± 10.1	22.4 ± 9.2	0.54	22.5 ± 9.8	22.4 ± 9.6	22.7 ± 10.5	22.2 ± 9.6	0.98

^1^Data are mean ± standard deviation (SD) or percent

^2^Obtained from ANOVA or chi-square test, where appropriate

**Fig. 1:**
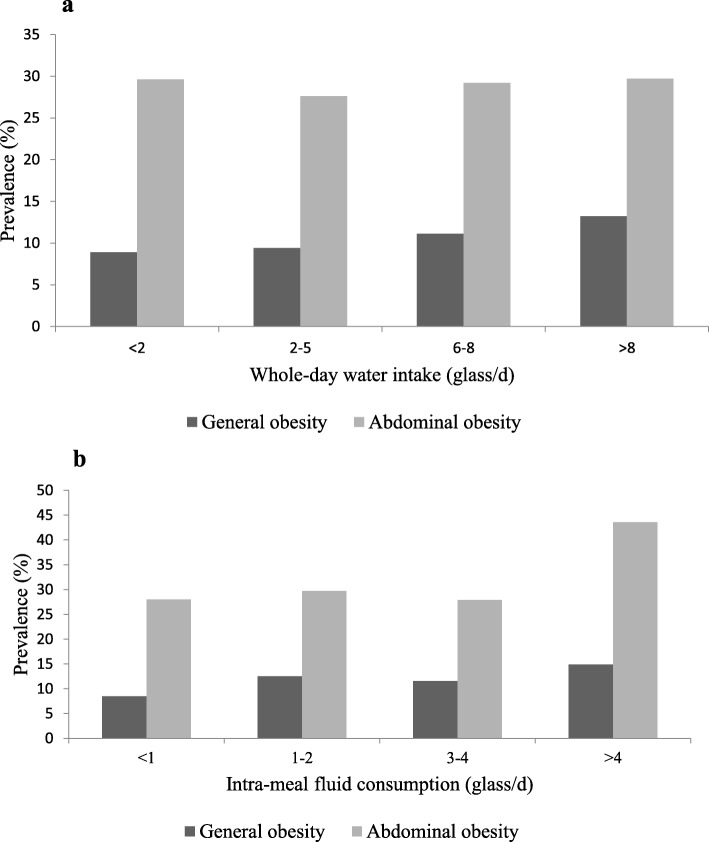
**a** Prevalence of general and abdominal obesity across categories of whole-day water intake. **b** Prevalence of general and abdominal obesity across categories of intra-meal fluid consumption

Prevalence of general and abdominal obesity across categories of whole-day water intake and intra-meal fluid consumption is shown in Fig. 1. Prevalence of general obesity in subjects who were taking > 8 glasses of water in a day was significantly higher than those who consumed 2 glasses of water daily (13.2 vs. 8.9%, *P = 0.01*). Compared to those who consumed less than one glass of fluid, individuals who consumed > 4 glasses of fluid between meals were more likely to be obese (14.9 vs. 8.5%, *P < 0.001*). This was also the case for abdominal obesity; such that individuals who consumed > 4 glasses of fluid between meals had higher prevalence of abdominal obesity than those who consumed one glass or less (43.6 vs. 28%, *P = 0.05*).

Crude and multivariable-adjusted means of anthropometric measures across categories of whole-day water intake and intra-meal fluid consumption are presented in Table 2. After taking potential confounders into account, subjects who consumed > 8 glasses of water during the day had higher means of weight (72.89 ± 0.84 vs. 64.95 ± 0.35 kg; *P < 0.001*), WC (89.51 ± 0.85 vs. 85.75 ± 0.34 cm; *P < 0.001*) and BMI (25.43 ± 0.25 vs. 24.36 ± 0.10 kg/m^2^; *P < 0.001*) compared with those who consumed less than two glasses of water. In addition, in the fully adjusted model, participants who consumed > 4 glasses of fluid between meals had greater means of weight (70.89 ± 1.89 vs. 66.57 ± 0.21 kg; *P < 0.001*), WC (90.82 ± 1.92 vs. 86.02 ± 0.21 cm; *P < 0.001*), and BMI (25.35 ± 0.56 vs. 24.46 ± 0.06 kg/m^2^; *P < 0.001*) compared with those who consumed less than one glass of fluid.

**Table 2:** Crude and multivariable-adjusted means for anthropometric measures across categories of whole-day water intake and intra-meal fluid consumption^1^

Whole-day water intake (glass/d)						Intra-meal fluid consumption (glass/d)				
	< 2	2–5	6–8	> 8	P-value	< 1	1–2	3–4	> 4	P-value
**Weight (kg)**										
Crude	66.00 ± 0.32	68.71 ± 0.25	71.85 ± 0.41	73.76 ± 0.80	< 0.001	67.56 ± 0.17	71.45 ± 0.27	72.45 ± 0.85	73.90 ± 1.46	< 0.001
Multivariable-adjusted^2^	64.95 ± 0.35	67.63 ± 0.24	70.73 ± 0.42	72.89 ± 0.84	< 0.001	66.57 ± 0.21	70.22 ± 0.35	71.54 ± 1.10	70.89 ± 1.89	< 0.001
**WC (cm)**										
Crude	86.03 ± 0.29	87.20 ± 0.20	88.71 ± 0.36	89.66 ± 0.71	< 0.001	86.52 ± 0.18	88.91 ± 0.29	89.70 ± 0.96	92.87 ± 1.57	< 0.001
Multivariable-adjusted^2^	85.75 ± 0.34	86.67 ± 0.24	87.48 ± 0.43	89.51 ± 0.85	< 0.001	86.02 ± 0.21	88.18 ± 0.35	88.98 ± 1.11	90.82 ± 1.92	< 0.001
**BMI (kg/m**^**2**^**)**										
Crude	24.63 ± 0.12	24.88 ± 0.09	25.43 ± 0.10	25.70 ± 0.21	< 0.001	24.76 ± 0.05	25.38 ± 0.08	25.13 ± 0.26	25.85 ± 0.45	< 0.001
Multivariable-adjusted^2^	24.36 ± 0.10	24.55 ± 0.07	25.03 ± 0.12	25.43 ± 0.25	< 0.001	24.46 ± 0.06	24.96 ± 0.10	24.77 ± 0.32	25.35 ± 0.56	< 0.001

^1^Data are mean ± standard error (SE)

^2^Adjusted for age, sex, total energy intake, education, physical activity, marital status, home ownership, eating rate, breakfast skipping, meal regularity and number of meals

Crude and multivariable-adjusted ORs and 95% CIs for general and abdominal obesity across categories of whole-day water intake and intra-meal fluid consumption are shown in Table 3. In the crude model, participants who were taking more than eight glasses of water in a day had 56% greater odds of general obesity (OR: 1.56; 95% CI: 1.11–2.19) compared with those who were taking less than two glasses of water in a day. After adjustment for potential confounders, this relationship was even strengthened (OR: 1.78; 95% CI: 1.08–2.94). Individuals with much water intake had no significant greater odds of abdominal obesity. In the crude model, subjects who were taking > 4 glasses of fluid between meals had 99% greater odds of abdominal obesity than those who consumed one glass or less (OR: 1.99; 95% CI: 1.16–3.41). However, the association became non-significant in the multivariable-adjusted model (OR: 1.65; 95% CI: 0.81–3.34). In the multivariable-adjusted model, subjects who consumed 1–2 glasses of fluids between meals had 34% greater odds of general obesity (OR: 1.34; 95% CI: 1.04–1.59) than those who consumed one glass or less.

**Table 3:** Crude and multivariable-adjusted ORs and 95% CIs for general and abdominal obesity across categories of whole-day water intake and intra-meal fluid consumption^1^

Whole-day water intake (glass/d)						Intra-meal fluid consumption (glass/d)				
	< 2	2–5	6–8	> 8	P-trend	< 1	1–2	3–4	> 4	P-trend
**General obesity**										
Crude	1.00	1.06 (0.88–1.27)	1.28 (1.02–1.61)	1.56 (1.11–2.19)	0.01	1.00	1.52 (1.29–1.79)	1.40 (0.91–2.16)	1.86 (0.97–3.57)	< 0.001
Multivariable-adjusted^2^	1.00	1.00 (0.75–1.33)	1.17 (0.82–1.68)	1.78 (1.08–2.94)	0.05	1.00	1.34 (1.04–1.59)	1.59 (0.85–2.96)	1.06 (0.32–3.49)	0.02
**Abdominal obesity**										
Crude	1.00	0.90 (0.79–1.03)	0.98 (0.82–1.16)	1.00 (0.75–1.33)	0.88	1.00	1.08 (0.95–1.23)	0.99 (0.69–1.43)	1.99 (1.16–3.41)	0.05
Multivariable-adjusted^2^	1.00	0.91 (0.76–1.08)	0.91 (0.72–1.13)	1.11 (0.78–1.60)	0.82	1.00	0.97 (0.82–1.14)	0.96 (0.61–1.52)	1.65 (0.81–3.34)	0.75

^1^Data are OR (95% CI)

^2^Adjusted for age, sex, total energy intake, education, physical activity, marital status, home ownership, eating rate, breakfast skipping, meal regularity and number of meals

## Discussion

This cross-sectional study examined whole-day water intake and intra-meal fluid consumption in relation to general and abdominal obesity among adults. We found that those who were taking more than eight glasses of water in a day and those who were consuming 1–2 glasses of fluids between meals had greater odds of general obesity.

The prevalence of obesity has been increasing in an alarming rate in the world [22]. Obesity is linked to several chronic conditions; therefore, investigating risk factors of obesity must be a priority for health care systems. In this study, we observed that those who were taking more than eight glasses of water in a day had greater odds of general obesity compared with those who were taking less than two glasses of water in a day; however, such finding was not observed between whole-day water intake and odds of abdominal obesity. Findings from a cross-sectional study revealed that obese adults consumed more plain water compared to normal-weight adults [23]. However, in contrast to our findings, some studies reported an inverse association between water intake and obesity. A cross-sectional study revealed that intake of water from foods, but not from beverages, was associated with a lower BMI and waist circumference [9]. A systematic review demonstrated that water intake was associated with reduced energy intake and therefore obesity prevention [5]. Some clinical trials showed no significant effect of water consumption on body weight [24–26]; however, in a clinical trial, consumption of 500 mL water before meals led to a greater weight loss compared to a hypocaloric diet in middle-aged and older adults [6]. It must be kept in mind that findings from clinical trials cannot be generalized to the routine life because these interventions are conducted in short time with high doses. Therefore, further evidence from observational studies will be important to make recommendations that are applicable to the general population. Although the information on the association between water intake and obesity are limited, the association of water consumption with other chronic diseases gained substantial attention in the literature [27, 28]. For instance, in a cross-sectional study on adults aged 20–84 years, high total water intake (> 4.3 L/day) was protectively linked with chronic kidney disease, however, cardiovascular disease was not associated with water consumption [27]. Higher total or plain water intake was also prospectively associated with slightly increased risk of mortality among women, but not men [28]. It must be noted that our study is of cross-sectional design and causality relationship between water intake and obesity cannot be inferred. Water consumption might help obese subjects to lose weight; however, such findings were not observed in general population [29]. Given the cross-sectional nature of this study, reverse causality is also possible: obese people were consuming much water in an attempt to lose weight.

Based on our findings, subjects who were consuming 1–2 glasses of fluids between meals had a greater chance of general obesity. It must be considered that we did not ask participants about type of beverages they were consuming with their meals. In Iranian culture, water ranks the first among common beverages consumed with meals [30]. However, some people consume yogurt drinks (namely Doogh) and colas with their meals. Previous studies have shown a significant direct association between sugar-sweetened beverages consumption and weight gain [31–33]. Based on prospective cohort studies, replacement of sugar-sweetened beverages with water resulted in a weight loss or a lower weight gain [12, 34]. Another cohort study showed that replacing of one sugar-sweetened soda beverage or beer by one serving of water per day was associated with a lower risk of obesity and greater weight loss [35]. However, our findings cannot be compared with these studies due to lack of information about type of beverages consumed with meals in the current study.

Energy intake might be associated with water intake, however, it must be kept in mind that energy intake has been considered as a covariate in this analysis. Therefore, any associations between dietary energy intake and exposure and outcome variables in this study have been controlled for.

Some strengths of this study include having a large sample size and considering several potential confounders into account. Several limitations should also be considered. First, due to cross-sectional nature of this study, causal relationship cannot be inferred. Therefore, further studies especially of prospective design are needed to confirm our findings. Second, although we controlled for several potential confounders, residual confounding cannot be excluded. To assess exposure, we used two simple questions. Although we used a pre-tested questionnaire, misclassification of study participants in terms of exposure variable cannot be ignored. In addition, obesity was defined based on self-reported data in this study. However, as mentioned above, our validation study revealed good correlations between self-reported anthropometric measures and actually-measured ones.

## Conclusion

In conclusion, we found that those who were taking more than eight glasses of water in a day and those who were consuming 1–2 glasses of fluids between meals had greater odds of general obesity.

## Data Availability

The dataset used and analyzed during the current study is available from the corresponding author on a reasonable request.

## Abbreviations

BMI: Body Mass Index
WC: Waist Circumferences
OR: Odds Ratio
CI: Confidence Interval
SEPAHAN: Study on the Epidemiology of Psychological, Alimentary Health and Nutrition project
FGDIs: Functional Gastrointestinal Disorders
DS-FFQ: Dish-based 106-item Semi-quantitative Food Frequency Questionnaire
NCEP: National Cholesterol Education Program
GPPAQ: General Practice Physical Activity Questionnaire.
